# A global planktic foraminifer census data set for the Pliocene ocean

**DOI:** 10.1038/sdata.2015.76

**Published:** 2015-12-08

**Authors:** Harry Dowsett, Marci Robinson, Kevin Foley

**Affiliations:** 1 US Geological Survey, Eastern Geology and Paleoclimate Science Center, Reston, Virginia 20192, USA

**Keywords:** Palaeoceanography, Biogeography, Palaeoecology

## Abstract

This article presents data derived by the USGS Pliocene Research, Interpretation and Synoptic Mapping (PRISM) Project. PRISM has generated planktic foraminifer census data from core sites and outcrops around the globe since 1988. These data form the basis of a number of paleoceanographic reconstructions focused on the mid-Piacenzian Warm Period (3.264 to 3.025 million years ago). Data are presented as counts of individuals within 64 taxonomic categories for each locality. We describe sample acquisition and processing, age dating, taxonomy and archival storage of material. These data provide a unique, stratigraphically focused opportunity to assess the effects of global warming on marine plankton.

## Background and Summary

The Pliocene (5.3 to 2.6 million years ago (Ma)), specifically the mid-Piacenzian^1,2^ (3.6 to 2.6 Ma), has been a focus of synoptic paleoclimate research for the past 25 years. The mid Piacenzian warm period (3.264 to 3.025 Ma) is the most recent time in Earth’s past that exhibited climates not unlike those projected for the end of the 21st century^3^. With widespread recognition by most experts that anthropogenic drivers are *extremely likely* to have been the dominant cause of observed warming since the mid-20th century^4^, and surface temperatures projected to rise over the 21st century under all emission scenarios^5^, understanding the Pliocene climate has taken on new importance. While not a direct analog to future climate conditions, there is much to learn about the magnitude and spatial distribution of change from this, in essence, natural climate laboratory.

Since 1988 the United States Geological Survey (USGS) has developed a large-scale data collection project: PRISM (Pliocene Research, Interpretation and Synoptic Mapping)^6^. Over this time PRISM has produced a series of ever more complex global paleoenvironmental reconstructions that provide probable estimates of Piacenzian ocean temperatures, sea level, sea ice extent, land ice distribution, vegetation or land cover, and elevation^6–11^. PRISM is the most detailed global reconstruction of Earth conditions for a past period of global warmth. The PRISM reconstructions serve two purposes: (1) they provide a conceptual model of mid-Piacenzian conditions and (2) they are formatted for use as boundary condition data sets as well as verification data for climate models.

Various elements of the PRISM reconstruction have been used in climate modelling experiments to test hypotheses and assess the performance of the models^9,11–15^. The latest PRISM reconstructions have been used by a number of climate modelling groups in the Pliocene Model Intercomparison Project (PlioMIP)^16^. PRISM research has documented a reduced pole to equator surface temperature gradient in both marine and terrestrial settings^13,17,18^, reduced longitudinal temperature gradients in the equatorial Pacific^19,20^, reduced sea ice and changes in ocean circulation^7,8^, elevated sea levels^21^ and major shifts in vegetation^22–24^.

**Figure 1:**
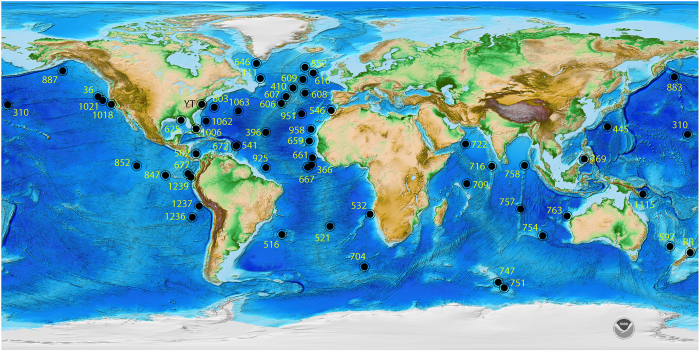
Map of sample locations from which Pliocene planktic foraminiferal census data were derived. Base map NOAA ETOPO1 global relief model^54^.

While the PRISM reconstruction has terrestrial, marine and cryospheric componets, the marine SST reconstruction has always been at the center of USGS PRISM work, and the faunal assemblage based SST data set has been the cornerstone of PRISM marine reconstructions. These SST reconstructions are based upon quantitative analysis of a large (>700,000 specimens) collection of mid-Piacenzian planktic foraminiferal data. Thus the PRISM planktic foraminifer collection, a census of individuals identified to species level from a global network of deep sea cores (Fig. 1) forms the basis for many of the PRISM paleoceanographic reconstructions^25–40^ and have been used by others in ecological niche modelling^41^ and analysis of diversity changes associated with global warming^42^. These data have been generated at the USGS since 1988, and additional data are being generated as part of the PRISM4 Paleoenvironmental reconstruction.

We present here raw faunal census data from 1,957 samples at 61 of our sites generated between 1988 and 2013 (Table 1 (available online only)). These data exist as counts of individual planktic forams placed into 64 taxonomic categories for each sample at each location. Samples are generally restricted to the Piacenzian Age as determined through a combination of magnetobiochronology and correlation of stratigraphic time series to the LR04 Marine Isotope Stages^43^.

## Methods

Our methodology for producing these data can be divided into three areas: chronology, sample acquisition and processing, and species identification.

### Chronology

All samples in the PRISM database are from the Pliocene Epoch and most fall within the Piacenzian Age. The PRISM ‘time slab’ or mid-Piacenzian Warm Period (mPWP) was originally defined as a 300 kyr interval of easily recognized warmth in the North Atlantic basin, centered on 3.0 Ma^44^. It was initially located in marine sections using magnetobiochronologic events. Over the past 25 years, events used to designate the mPWP have changed, and the geologic time scale used to calibrate those events has been refined and revised. Some newer sequences are dated by tuning stratigraphic records to known insolation changes caused by cyclical variations of the Earth’s orbit. The interval of time, the mPWP, the last time Earth experienced warming on the scale projected for the end of the 21st Century, has remained the same throughout the project.

**Figure 2:**
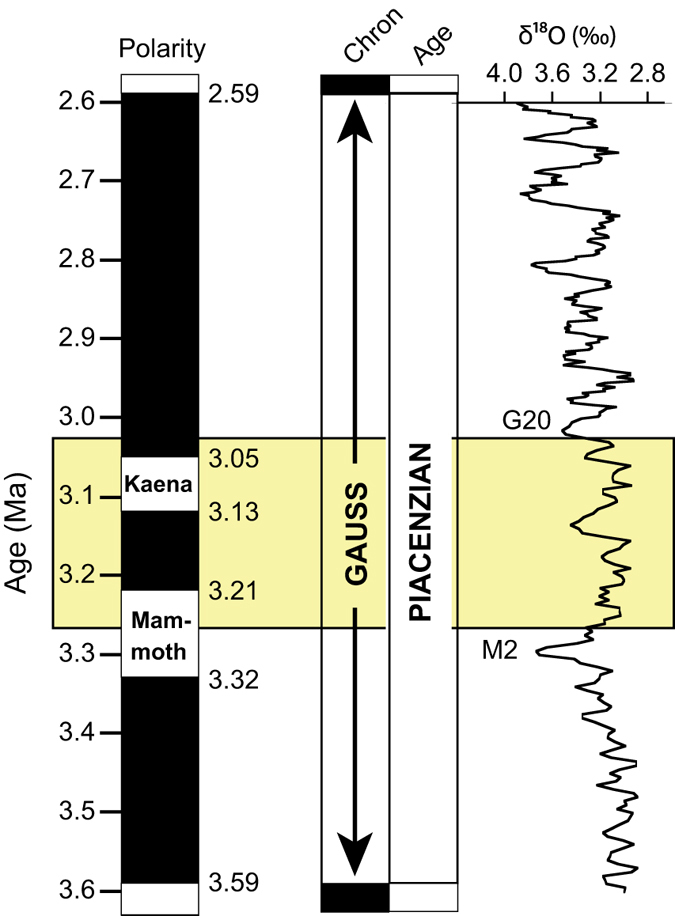
Chronostratigraphic framework for the mPWP. Position of the PRISM ‘time slab’ or mPWP (yellow shading) with respect to paleomagnetic stratigraphy (Gauss Chron) and the LR04 stack^43^ of benthic δ^18^O records. The mPWP extends from 3.264 Ma (within the Mammoth subchron) to 3.025 Ma (just above Kaena subchron)^8^ Marine Isotope stages G20 and M2 are identified. Black is normal polarity, white reversed polarity.

The mPWP is presently defined as the period between the transition of marine isotope stages (MIS) M2/M1 (3.264 Ma) and G21/G20 (3.025 Ma) in the middle part of the Gauss Polarity Chron (Fig. 2)^8^. This interval ranges from C2An2r (Mammoth reversed polarity) to near the bottom of C2An1 (just above Kaena reversed polarity). This 239 kyr time slab correlates in part to planktonic foraminiferal zones PL3 (*Sphaeroidinellopsis seminulina* Highest Occurrence Zone), PL4 (*Dentoglobigerina altispira* Highest Occurrence Zone) and PL5 (Atlantic) (*Globorotalia miocenica* Highest Occurrence Zone) or PL5 (Indo-Pacific) (*Globorotalia pseudomiocenica* Highest Occurrence Zone)^45^.

Age determinations presented here are based on the best available data at the time of original investigation, however data contained in this archive were generated over a period of 25 years (Supplementary File 1). Age models for most core sites contained in the PRISM planktic foram census data set are based upon biochronology (calibrated first and last occurrence events for faunal and floral taxa), magnetostratigraphy (dated paleomagnetic reversals), magnetobiochronology (combination of biochronologic and paleomagnetic calibrated events), tephrachronology (radiometrically dated ash beds), graphic correlation (Shaw’s method of correlation via a magnetobiochronological model) or astronomical tuning (direct or indirect correlation of time series [usually δ^18^O] to orbital forcing). Astronomical tuning was applied to these sequences: (607, 610, 659, 763, 806, 847, 852, 925, 1014, 1237, and 1239).

Due to the inconsistencies of calibrated datums both regionally and over the time period these cores were analyzed, as well as the many versions of geological time scales, users are urged to research and develop their own age models for these sites. Comparison of samples from one location to another, based upon provided ages, will result in diachronous correlations. Therefore, users are advised to consult the most current paleontological and chronological data for these sites.

### Sample acquisition and processing

The majority of samples come from cores raised by DSDP, ODP, and from a number of outcrops on land. For marine cores, 10–20 cc samples were removed from a split core using a cylindrical plug and sealed in a plastic bag for transport to the USGS. Outcrop samples were retrieved using a hand shovel and rock hammer to obtain approximately 50–500 g of sediment. Samples were placed in plastic bags for transport to the USGS. In the lab, samples were oven dried at ≤50 °C, and then soaked and agitated in water with ~2 ml of dilute sodium hexametaphosphate solution (5 g to 1 l water) for 1–2 h. Samples were then washed over a 63 μ or 150 μ sieve until clean. Samples were then oven dried at ≤50 °C, then dry-sieved to concentrate the ≥150 μ fraction. The ≥150 μ fraction was placed in a sample splitter and split until ~300 planktic foraminifera specimens were obtained. There is a 0.05 probability that we failed to detect a taxon represented by 3 individuals (1%) in a population of 300 individuals^46^. Reducing the probability to 0.01 would require counting an additional 200 specimens. Census counts are labor-intensive, and using 300 specimens is common practice in studies similar to ours. In samples that did not contain 300 planktic foraminifer specimens, all planktic foraminifers were counted. Specimens were placed on a Plummer slide (60 cell faunal micro slide) for identification and sorting into 64 possible taxonomic categories (Supplementary File 2). Foraminifers were manipulated with a fine (00000) paintbrush under an incident light microscope and fixed to the slide using a weak, water-soluble glue.

### Species identification and archival samples

Individual specimens were identified to species level following taxonomic concepts of Parker^47,48^, Blow^49^, and Dowsett and Robinson^30^ (Supplementary File 2). All counts were generated by the PRISM Project; Data published by others are not included in this release.

Foraminifers were grouped by species and fixed in place on slides, and additional washed residue (when available) for all samples shown in the global planktic foraminifer census database, are physically archived at the US Geological Survey in Reston, Virginia, USA.

## Data Records

The census of 593,676 individuals identified to species level in 1,957 Pliocene age ocean sediment samples is accessible at National Climate Data Center (NCDC) (Data Citation 1: Global Planktic Foraminifer Census Data Set for the Pliocene Ocean https://www.ncdc.noaa.gov/paleo/study/19281). The data for each sample consist of location information (name and geographic coordinates), sample number, position in stratigraphic sequence (depth below sea floor for sediment cores and height above base of land section for terrestrial outcrops), age, and number of individuals assigned to each of 64 taxonomic categories (see Supplementary Files 1 and 2).

## Technical Validation

Micropaleontological techniques for processing and sorting individual foraminifer tests into species are well documented and standardized in the paleoceanographic community^50–52^. Large projects, similar to PRISM, have generated planktic foraminiferal census data and an important factor for these studies has been maintaining internal consistency in identifications^53^. We maintain consistency and avoid variation in identification of species by having a small number of individuals with the same taxonomic concepts identify all specimens. We further reduce the possibility of taxonomic drift by having all identifications checked by one micropaleontologist associated with the project since its inception. We consider this taxonomic consistency a primary strength of our data.

## Additional Information

Table 1 is only available in the online version of this paper.

**How to cite this article:** Dowsett, H. *et al.* A global planktic foraminifer census data set for the Pliocene ocean. *Sci. Data* 2:150076 doi: 10.1038/sdata.2015.76 (2015).

## Supplementary Material



Supplementary Information

Supplementary Information

## Figures and Tables

**Table 1:** Samples, subjects, and data outputs

**Locality**	**Number of samples**	**Temporal range estimate**	**Protocol 1**	**Protocol 2**	**Data**
DSDP 36	18	3.510–3.023	Foraminiferal census	Species counts	dsdp36.txt
DSDP 111	20	4.000–2.000	Foraminiferal census	Species counts	dsdp111a.txt
DSDP 310	29	3.205–1.603	Foraminiferal census	Species counts	dsdp310.txt
DSDP 366	11	6.181–2.642	Foraminiferal census	Species counts	dsdp366a.txt
DSDP 396	14	3.817–3.070	Foraminiferal census	Species counts	dsdp396.txt
DSDP 410	12	3.330–2.693	Foraminiferal census	Species counts	dsdp410.txt
DSDP 445	23	4.077–2.824	Foraminiferal census	Species counts	dsdp445.txt
DSDP 502	70	3.600–2.875	Foraminiferal census	Species counts	dsdp502a.txt
DSDP 516	26	3.490–2.050	Foraminiferal census	Species counts	dsdp516a.txt
DSDP 521	21	3.232–2.887	Foraminiferal census	Species counts	dsdp521.txt
DSDP 532	28	3.709–2.492	Foraminiferal census	Species counts	dsdp532.txt
DSDP 541	33	3.420–2.330	Foraminiferal census	Species counts	dsdp541.txt
DSDP 546	34	3.535–1.961	Foraminiferal census	Species counts	dsdp546.txt
DSDP 552	124	5.045–2.388	Foraminiferal census	Species counts	dsdp552a.txt
DSDP 592	68	3.366–2.466	Foraminiferal census	Species counts	dsdp592.txt
DSDP 603	53	3.460–1.710	Foraminiferal census	Species counts	dsdp603c.txt
DSDP 606	120	4.423–2.301	Foraminiferal census	Species counts	dsdp606.txt
DSDP 607	31	3.943–2.303	Foraminiferal census	Species counts	dsdp607.txt
DSDP 608	39	3.156–2.838	Foraminiferal census	Species counts	dsdp608.txt
DSDP 609	34	4.412–2.268	Foraminiferal census	Species counts	dsdp609b.txt
DSDP 610	33	4.864–2.314	Foraminiferal census	Species counts	dsdp610a.txt
ODP 625	25	3.331–2.919	Foraminiferal census	Species counts	odp625b.txt
ODP 646	72	5.130–3.361	Foraminiferal census	Species counts	odp646b.txt
ODP 659	27	3.535–3.020	Foraminiferal census	Species counts	odp659a.txt
ODP 661	34	3.330–2.969	Foraminiferal census	Species counts	odp661a.txt
ODP 667	35	3.557–2.378	Foraminiferal census	Species counts	odp667a.txt
ODP 672	39	4.261–2.635	Foraminiferal census	Species counts	odp672a.txt
ODP 677	28	3.307–2.953	Foraminiferal census	Species counts	odp677a.txt
ODP 704	32	3.441–3.125	Foraminiferal census	Species counts	odp704a.txt
ODP 709	21	3.346–3.070	Foraminiferal census	Species counts	odp709c.txt
ODP 716	23	3.332–2.952	Foraminiferal census	Species counts	odp716b.txt
ODP 722	11	3.283–2.866	Foraminiferal census	Species counts	odp722a.txt
ODP 747	17	3.300–2.600	Foraminiferal census	Species counts	odp747a.txt
ODP 751	6	3.100–2.500	Foraminiferal census	Species counts	odp751a.txt
ODP 754	30	3.350–2.710	Foraminiferal census	Species counts	odp754a.txt
ODP 757	12	3.350–2.910	Foraminiferal census	Species counts	odp757b.txt
ODP 758	30	3.303–2.976	Foraminiferal census	Species counts	odp758a.txt
ODP 763	29	3.320–2.904	Foraminiferal census	Species counts	odp763a.txt
ODP 769	26	5.300–2.310	Foraminiferal census	Species counts	odp769b.txt
ODP 847	25	3.305–2.958	Foraminiferal census	Species counts	odp847c.txt
ODP 852	12	3.823–2.837	Foraminiferal census	Species counts	odp852b.txt
ODP 883	72	3.231–3.087	Foraminiferal census	Species counts	odp883bc.txt
ODP 887	51	3.851–2.820	Foraminiferal census	Species counts	odp887ac.txt
ODP 925	47	3.342–2.851	Foraminiferal census	Species counts	odp925b.txt
ODP 951	32	3.304–2.955	Foraminiferal census	Species counts	odp951a.txt
ODP 958	34	3.300–2.970	Foraminiferal census	Species counts	odp958a.txt
ODP 1006	34	3.300–2.970	Foraminiferal census	Species counts	odp1006a.txt
ODP 1018	30	3.070–2.963	Foraminiferal census	Species counts	odp1018a.txt
ODP 1021	10	2.725–2.684	Foraminiferal census	Species counts	odp1021c.txt
ODP 1062	34	3.300–2.970	Foraminiferal census	Species counts	odp1062b.txt
ODP 1063	31	3.300–2.970	Foraminiferal census	Species counts	odp1063a.txt
ODP 1115	23	3.325–2.938	Foraminiferal census	Species counts	odp1115b.txt
ODP 1236	10	3.200–2.760	Foraminiferal census	Species counts	odp1236a.txt
ODP 1237	19	3.421–2.931	Foraminiferal census	Species counts	odp1237c.txt
ODP 1239	33	3.272–2.959	Foraminiferal census	Species counts	odp1239b.txt
Deep Creek (Yorktown)	26	3.250–3.250	Foraminiferal census	Species counts	yorktown.txt
Rangitikei	49	3.350–3.250	Foraminiferal census	Species counts	rangitikei.txt
