# Supplementary material for: A global planktic foraminifer census data set for the Pliocene ocean
Source: Sci Data. 2015 Dec 8;2:150076. doi: 10.1038/sdata.2015.76 (PMC4672679; doi:10.1038/sdata.2015.76)
Supplement: Supplementary Information [file sdata201576-s2.doc]

**A global planktic foraminifer census data set for the Pliocene ocean**

**Harry Dowsett^1^, Marci Robinson^1^, Kevin Foley^1^**

**Affiliation**

1. U.S. Geological Survey, Eastern Geology and Paleoclimate Science Center, Reston, VA, USA

corresponding author: Harry Dowsett (hdowsett@usgs.gov)

### Supplementary Information

This Supplementary File presents the taxonomic categories used to generate the census data by way of a species list. The first entry contains the full species name and original author. The second entry gives the original reference for the species. We include a bibliography of the publications in which the original references can be found.

**Species List**

*Candeina nitida* d’Orbigny

*Candeina nitida* D’ORBIGNY 1839, p. 107, pl. 2, figs. 27-28

*Dentoglobigerina altispira* (Cushman and Jarvis)

*Globigerina altispira* CUSHMAN and JARVIS 1936, p. 5, pl. 1, figs.

13a-c

*Dentoglobigerina baroemoensis* (LeRoy)

*Globigerina baroemoensis* LEROY 1939, p. 263, pl. 6, figs. 1-2

*Globigerina apertura* Cushman

*Globigerina apertura* CUSHMAN 1918, p. 57, pl. 12, figs. 8a-c.

*Globigerina bulloides* d’Orbigny

*Globigerina bulloides* D’ORBIGNY 1826, p. 277,. 1, figs. 1-4

*Globigerina calida* Parker

*Globigerina calida* PARKER 1962, p. 221, pl. 1, figs 9-13, 15

*Globoquadrina conglomerata* (Schwager)

*Globigerina conglomerata* SCHWAGER 1866, p. 255, pl. 7, fig. 113

*Globigerina decoraperta* Takayanagi and Saito

*Globigerina druryi* Akers *decoraperta* TAKAYANAGI and SAITO,

1962, p. 85, pl. 28, figs 10a-c.

*Globigerina digitata* Brady

*Globigerina digitata* BRADY 1879, vol. 19, p. 286 (no figures); BRADY 1884, p. 599, pl. 80, figs. 6-10.

*Globigerina eamesi* Blow

*Globigerina eamesi* BLOW 1959, p. 176, pl. 9, figs. 39a-c

*Globigerina falconensis* Blow

*Globigerina falconensis* BLOW 1969, p.177, pl. 9, figs. 40a-c, 41

*Globigerina incisa* (Bronnimann and Resig)

*Globorotalia incisa* BRONNIMANN and RESIG 1971, p. 1278-1279,

pl. 45, figs. 5,7, pl. 46, figs. 1-8.

*Globigerina nepenthes* Todd

*Globigerina nepenthes* TODD 1957, p. 3-1, figs. 7a-7b.

*Globigerina obesa* (Bolli)

*Globorotalia obesa* BOLLI 1957, p. 119, pl. 29, figs. 2a-3

*Globigerina praedigitata* Parker

*Globigerina praedigitata* PARKER 1967, p. 151, pl. 19, figs. 5–8.

*Globigerina rubescens* Hofker

*Globigerina rubescens* HOFKER 1956, p. 234, pl. 35, figs. 18-21

*Globigerina umbilicata* Orr and Zaitzeff

*Globigerina umbilicata* ORR and ZAITZEFF 1971, p. 18, pl. 1, figs. 1-3

*Globigerina woodi* Jenkins

*Globigerina woodi* JENKINS 1960, p. 352, pl. 2, figs. 2a-2c.

*Globigerinella aequilateralis* (Brady)

*Globigerina aequilateralis* BRADY 1879, p. 285 (figs. in Brady 1884,

pl. 80, figs. 18-21).

*Globigerinella siphonifera* (d’Orbigny)

*Globigerina siphonifera* D’ORBIGNY 1839, p. 83, pl. 4, figs. 15-18.

*Globigerinella pseudobesa* (Salvatorini)

*Turborotalita pseudobesa* SALVATORINI 1966, p.10, pl. 2, figs.

6a-15.

*Globigerinita glutinata* (Egger)

*Globigerina glutinata* EGGER 1893, p. 371, pl. 13, figs. 19-21

*Globigerinoides conglobatus* Brady

*Globigerinoides conglobatus* BRADY 1879, p. 28b.

*Globigerinoides fistulosus* (Schubert)

*Globigerina fistulosa* SCHUBERT 1910, p. 323, text fig. 1.

*Globigerinoides obliquus* Bolli

*Globigerinoides obliquus* BOLLI 1957, p. 113, pl. 25, figs. 10a-c

*Globigerinoides obliquus extremus* Bolli and Bermudez

*Globigerinoides obliquus extremus* BOLLI and BERMUDEZ 1965, p.

139, pl. 1, figs. 10-12

*Globigerinoides ruber* (d’Orbigny)

*Globigerina rubra* D’ORBIGNY 1839, p. 82, pl. 4, figs. 12-14

*Globigerinoides sacculifer* (Brady)

*Globigerina sacculifera* BRADY 1877, p. 164, pl. 9, figs. 7–10.

*Globigerinoides tenellus* Parker

*Globigerinoides tenellus* PARKER 1958, p.280, pl. 6, figs. 7-11.

*Globoquadrina venezuelana* (Hedberg)

*Globigerina venezuelana* HEDBERG 1937, p. 681, pl. 92, figs. 7a-b.

*Globorotalia conomiozea* Kennett

*Globorotalia conomiozea* KENNETT 1966, p. 235, figs. 10a-c.

*Globorotalia crassaformis* (Galloway and Wissler)

*Globigerina crassaformis* GALLOWAY and WISSLER 1927, p. 41, pl.

7, fig. 12.

*Globorotalia crassula* (Cushman and Stewart)

*Pulvinulina crassa* BRADY 1884, p. 694, pi. 103, figs. 11-12

*Globorotalia hirsuta* (d’Orbigny)

*Rotalina hirsuta* D’ORBIGNY 1839, p. 131, pl. 1, figs. 34–36.

*Globorotalia inflata* (d’Orbigny)

*Globorotalia inflata* D’ORBIGNY 1839 *in* Barker-Webb and Berthelot, vol. 2, pt. 2, p. 134, pl. 2, figs. 7-9.

*Globorotalia limbata* (Fornasini)

*Rotalia limbata* FORNASINI 1902, p. 56, fig. 55

*Globorotalia margaritae* Bolli and Bermudez

*Globorotalia margaritae* BOLLI and BERMUDEZ 1965, p. 138, pl. 1,

figs. 1-9.

*Globorotalia menardii* (Parker, Jones, and Brady)

*Rotalia menardii* PARKER, JONES, and BRADY1865, p. 20, pl. 3, fig. 81

*Globorotalia multicamerata* Cushman and Jarvis

*Globorotalia multicamerata* CUSHMAN and JARVIS 1930, p. 367, pl. 34, figs. 8a-c

*Globorotalia praepumilio* (Parker)

*Globanomalina praepumilio* PARKER 1967, p. 148, pl. 18, fig. 3

*Globorotalia pumilio* Parker

*Globorotalia pumilio* PARKER 1962, p. 238, pl. 6, figs. 2-3

*Globorotalia puncticulata* (Deshayes)

*Globigerina puncticulata* DESHAYES 1832, p. 170 (figs. in Fornasini,

1899, p. 210, fig. 5)

*Globorotalia scitula* (Brady)

*Pulvinulina scitula* BRADY 1882, p. 27, pl. 5, fig. 5

*Globorotalia tosaensis* Takayanagi and Saito

*Globorotalia tosaensis* TAKAYANAGI and SAITO 1962, p. 81, pl. 28, figs. 11a-12c

*Globorotalia truncatulinoides* (d’Orbigny)

*Rotalia truncatulinoides* D’ORBIGNY 1839 *in* Barker-Webb and Berthelot, vol. 2, pt. 2, p. 132, pl. 2, figs. 25-27.

*Globorotalia tumida* (Brady)

*Pulvinulina menardii* (d’Orbigny) var. *tumida* BRADY 1877, pl. 103,

figs. 4-6.

*Globorotalia ungulata* Bermudez

*Globorotalia ungulata* BERMUDEZ 1960, p. 1304, pl. 15, figs. 6a-b

*Globorotaloides hexagona* (Natland)

*Globigerina hexagona* NATLAND 1938, p. 149, pl. 7, figs. 1a-c

*Neogloboquadrina acostaensis* (Blow)

*Globorotalia acostaensis* BLOW 1959, p. 208, pl. 17, figs. 106a-106c.

*Neogloboquadrina atlantica* Berggren

*Neogloboquadrina atlantica* BERGGREN 1972, pl. 1, figs. 7-9.

*Neogloboquadrina continuosa* (Blow)

*Globorotalia opima* BOLLI subspecies *continuosa* BLOW 1959, p. 218, pl. 19, figs. 125a-c

*Neogloboquadrina dutertrei* (d’Orbigny)

*Neogloboquadrina dutertrei* D’ORBIGNY 1826, p. 277, no. 6

*Neogloboquadrina humerosa* (Takayanagi and Saito)

*Globorotalia humerosa* TAKAYANAGI and SAITO 1962, p. 78, pl. 28,

figs. 1a-2b.

*Neogloboquadrina pachyderma* (Ehrenberg)

*Aristopira pachyderma* EHRENBERG 1861, p. 276-277, 303.

*Orbulina universa* d’Orbigny

*Orbulina universa* D’ORBIGNY 1839, p. 3, pl. 1, fig. 1

*Pulleniatina obliquiloculata* (d’Orbigny)

*Pullenia sphaeroides* (d’Orbigny) var. *obliquiloculata* PARKER and

JONES 1865, p. 368, pl. 19, figs. 4a–b.

*Sphaeroidinellopsis seminulina* (Schwager)

*Globigerina seminulina* SCHWAGER 1866, p. 256, pl. 7, fig. 112.

*Turborotalita quinqueloba* (Natland)

*Globigerina quinqueloba* NATLAND 1938, p. 149, pl. 6, figs. 7a-c.

**Taxonomic References**

Barker-Webb, P., and Berthelot, S., 1840, *Histoire naturelle des Iles Canaries* Paris, Belthune, v.2.

Berggren, W.A., 1972, Cenozoic biostratigraphy and paleobiogeography of the North Atlantic: *Initial Reports of the Deep Sea Drilling Project*, v. 12, n. 14, p. 965-1001.

Blow, W.H., 1959, Age, correlation and biostratigraphy of the upper Tocuyo (San Lorenzo) and Pozón Formations, eastern Falcon, Venezuela: *Bulletins of American Paleontology*, v. 39, n. 178, p. 67-252.

Blow, W.H., 1969, Late middle Eocene to recent planktonic foraminiferal biostratigraphy, in Bronniman, P., and Renz, H.H., eds., *Proceedings of the 1st International Conference on Planktonic Microfossils* Leiden, The Netherlands, Brill, E. J. , p. 199-422.

Bolli, H., 1957, Planktonic foraminifera from the Oligocene-Miocene Cipero and Lengua Formations of Trlnidad, B.W.I.: *U.S. National Museum Bulletin*, n. 215, p. 97-103.

Bolli, H.M., and Bermudez, P.J., 1965, Zonation based on Planktonic foraminifera of Middle Miocene to Pliocene warm-water sediments: *Bol. Inform. Asoc. Venezolana Geol. Min. Petr*, v. 8, p. 121-149.

Brady, H.B., 1877, II.—Supplementary Note on the Foraminifera of the Chalk (?) of the New Britain Group, *Geological Magazine (Decade II)*, v. *4*, N. 12, p. 534-536.

Brady, H.B., 1879, Memoirs: Notes on some of the Reticularian Rhizopoda of the" Challenger" Expedition: *Quarterly Journal of Microscopical Science*, v. 2, n. 73, p. 20-63.

Brady, H.B., 1882, Report on the Foraminifera: in TIZARDS and MURRAY, Exploration of the Faroé Channel during the Summer of 1880: Her Majesty’s Hired Ship “Knight Errant”: *Royal Society of Edinburgh, Proc*, v. 11, p. 1880-1882.

Brady, H.B., 1884, Report on the foraminifera dredged by H.M.S. Challenger during the years 1873-1876, Report of the scientific results of the voyage of H.M.S. Challenger, 1873-1876, *Zoology*, v. 9: London, p. 1-814.

Brönnimann, P., and Resig, J., 1971, A Neogene globigerinacean biochronologic time-scale of the southwestern Pacific: *Initial Reports of the Deep Sea Drilling Project*, v. 7, p. 1235-1469.

Cushman, J.A., 1918, Some Pliocene and Miocene foraminifera of the coastal plain of the United States: *U.S. Geological Survey Bulletin*, n. 676, p. 1-100.

Cushman, J.A., and Jarvis, P., 1930, Miocene foraminifera from Buff Bay, Jamaica: *Journal of Paleontology*, p. 353-368.

Cushman, J.A. and Jarvis, P.W., 1936, Three new foraminifera from the Miocene Bowden Marl of Jamaica, *Contrib. Cushman Lab. Foraminiferal Res*, v. *12*, p. 3-5.

Cushman, J.A., Stewart, R.E., and Stewart, K.C., 1930, Tertiary foraminifera from Humboldt County, California: A preliminary survey of the fauna: *Transactions of the San Diego Society of Natural History*, v. 6, n. 2, p. 41-94.

d'Orbigny, A.D., 1826, Tableau methodique de la classes des Cephalopodes: *Annales Sciences Naturelles*, v. 7, p. 277.

d'Orbigny, A.D., 1839, Foraminiferes, in Sagra, R.D.L., ed., *Histoire physique, politique et naturelle de I'lle de Cuba*: Paris, A. Bertrand, p. 1-224.

Deshayes, G.P., 1832, Encyclopédie Méthodique. *Histoire Naturelle des Vers*, Tome 2, Paris, p. 1-594.

Ehrenberg, C.G., 1861, Elemente des tiefen Meertesgrundes in Mexikanischen Golfstrome bei Florida; über die Tiefgrund-Verhåltnisse des Oceans am Eingage der Davisstrasse und bei Island, *Monatsbericht, Koniglich-preussische Akademie der Wissenschaften zu Berlin*, p. 222-240; 275-315

Fornasini, C., 1902, Sinossi metodica dei Foraminiferi sin qui rinvenuti nella sabbia del Lido di Rimini, *Academia de la scienze di Bologna*.

Galloway, J.J., and Wissler, S.G., 1927, Pleistocene foraminifera from the Lomita Quarry, Palos Verdes Hills, California: *Journal of Paleontology*, p. 35-87.

Hedberg, H.D., 1937, Foraminifera of the middle Tertiary Carapita Formation of northeastern Venezuela, *Journal of Paleontology*, p. 661-697.

Hofker, J., 1956, Foraminifera Dentata: Foraminifera of Santa Cruz and Thatch-Island, Virginia-Archipelago, West-Indies, *Copenhagen Univ., Zool. Mus., Spolia (Skrifter)*, v. 15, p. 234.

Jenkins, D.G., 1960, Planktonic foraminifera from the Lakes Entrance oil shaft, Victoria, Australia, *Micropaleontology*, p. 345-371.

Kennett, J.P., 1966, The *Globorotalia crassaformis* bioseries in north Westland and Marlborough, New Zealand, *Micropaleontology*, p. 235-245.

LeRoy, L.W., 1939, Some Small Foraminifera, Ostracoda and Otoliths from the Neogene (" Miocene") of the Rokantapanoeli Area, Central Sumatra, *Naturkuundig tijdschrift voor Nederlandsch-Indie*, v. 99, p. 215-296.

Natland, M.L., 1938, New species of foraminifera from off the west coast of North America and from the later Tertiary of the Los Angeles Basin, *Bulletin, Scripps Institution Oceanography, Technical Series*, v. 4, p. 137-164.

Orr, W.N., and Zaitzeff, J.B., 1971, A new planktonic foraminiferal species from the California Pliocene: *Journal of Foraminiferal Research*, v. 1, n. 1, p. 17-19.

Parker F.L., 1958, Eastern Mediterranean foraminifera, Sediment Cores from the Mediterranean Sea and the Red Sea. *Rep. Swedish Deep-Sea Exp*., v. 8, n. 4, p. 219-285.

Parker, F.L., 1962, Planktonic foraminiferal species in Pacific sediments: *Micropaleontology*, v. 8, n. 2, p. 219-254.

Parker, F.L., 1967, Late Tertiary biostratigraphy (planktonic foraminifera) of tropical Indo-Pacific deep-sea cores: *Bulletin of American Paleontology*, v. 52, p. 115-208.

Parker, W.K., Jones, T., and Brady, H.B., 1865, IV—On the nomenclature of the Foraminifera, *Journal of Natural History*, V. *16*, N. 91, p. 15-41.

Schubert, R.J., 1910, Ueber Foraminiferen und einen Fischotolithen aus dem fossilen Globigerinenschlamm von Neu-Guinea, *Geol. Reichsanst*., p. 318-328.

Schwager, C., 1866, Fossile Foraminiferen von Kar Nikobar, Bd. 2, *Geol. Theil,* p. 187-268.

Takayanagi, Y. and Saito, T., 1962, Planktonic foraminifera from the Nobori Formation, Shikoku, Japan, *Science Reports, Tohoku University, Sendai, Japan, 2^nd^ Ser. (Geol.), Spec. V*., N. 5, p. 67-106.

Todd, R., 1957, Smaller foraminifera, Geology of Saipan, Mariana Islands, Part, 3, Paleontology, *U.S. Geological Survey Professional Paper* 280-H, p. 265-320.
