# Supplementary material for: A global planktic foraminifer census data set for the Pliocene ocean
Source: Sci Data. 2015 Dec 8;2:150076. doi: 10.1038/sdata.2015.76 (PMC4672679; doi:10.1038/sdata.2015.76)
Supplement: Supplementary Information [file sdata201576-s3.doc]

**A global planktic foraminifer census data set for the Pliocene ocean**

**Harry Dowsett^1^, Marci Robinson^1^, Kevin Foley^1^**

**Affiliation**

1. U.S. Geological Survey, Eastern Geology and Paleoclimate Science Center, Reston, VA, USA

corresponding author: Harry Dowsett (hdowsett@usgs.gov)

### Supplementary Information

This Supplementary File provides additional information on the method and types of data used to derive original ages for all samples. Additionally, primary references containing information on the origin of the ages are provided. Methods include biochronology: calibrated first and last occurrence events for faunal and floral taxa; magnetostratigraphy: dated paleomagnetic reversals; magnetobiochronology: combination of biochronologic and paleomagnetic calibrated events; tephrachronology: radiometrically dated ash beds; graphic correlation: Shaw’s method of correlation via a magnetobiochronological model; astronomical tuning: direct or indirect correlation of time series (usually δ^18^O) to orbital forcing.

Due to the inconsistencies of calibrated datum both regionally and over the time period these cores were analyzed, as well as the many differences in geological time scales (e.g., Berggren et al., 1985; Berggren et al., 1995; Shackleton et al. 1995; Lisiecki and Raymo, 2005; Gradstein et al., 2012) users are urged to research and develop their own age models for these sites. Comparison of samples from one location to another, based upon provided ages, will result in diachronous correlations.

**Age Model Information**

| **Locality** | **Dating method** | **Type of paleontological data** | **Primary references** |
| --- | --- | --- | --- |
| [DSDP 111A Data](http://www1.ncdc.noaa.gov/pub/data/paleo/contributions_by_author/foley2015/foley2015-dsdp111a.txt) | biochronology | planktonic foraminifers | Shipboard Scientific Party (1972); Berggren (1972) |
| [DSDP 310 Data](http://www1.ncdc.noaa.gov/pub/data/paleo/contributions_by_author/foley2015/foley2015-dsdp310.txt) | magnetobiochronology | planktic foraminifers, radiolaria, diatom | Keller (1978 |
| [DSDP 36 Data](http://www1.ncdc.noaa.gov/pub/data/paleo/contributions_by_author/foley2015/foley2015-dsdp36.txt) | biochronology, tephrachronology | planktic foraminifers | Poore (1999) |
| [DSDP 366A Data](http://www1.ncdc.noaa.gov/pub/data/paleo/contributions_by_author/foley2015/foley2015-dsdp366a.txt) | biochronology | nannofossils | Shipboard Scientific Party (1978) |
| [DSDP 396 Data](http://www1.ncdc.noaa.gov/pub/data/paleo/contributions_by_author/foley2015/foley2015-dsdp396.txt) | biochronology | planktic foraminifers, nannofossils | Shipboard Scientific Party (1979) |
| [DSDP 410 Data](http://www1.ncdc.noaa.gov/pub/data/paleo/contributions_by_author/foley2015/foley2015-dsdp410.txt) | graphic correlation | planktic foraminifers, nannofossils | Poore (1978), Poore et al. (1978), Dowsett (1989a) |
| [DSDP 445 Data](http://www1.ncdc.noaa.gov/pub/data/paleo/contributions_by_author/foley2015/foley2015-dsdp445.txt) | graphic correlation | planktic foraminifers, nannofossils | Dowsett (1989a,b); Dowsett and Robinson (1998) |
| [DSDP 502A Data](http://www1.ncdc.noaa.gov/pub/data/paleo/contributions_by_author/foley2015/foley2015-dsdp502a.txt) | magnetostratigraphy, astronomical tuning, graphic correlation | planktic foraminifers, nannofossils | Kent and Spariosu (1982), Dowsett (1989a) |
| [DSDP 516A Data](http://www1.ncdc.noaa.gov/pub/data/paleo/contributions_by_author/foley2015/foley2015-dsdp516a.txt) | graphic correlation, magnetostratigraphy | planktic foraminifers, nannofossils | Dowsett (1989a) |
| [DSDP 521 Data](http://www1.ncdc.noaa.gov/pub/data/paleo/contributions_by_author/foley2015/foley2015-dsdp521.txt) | graphic correlation, magnetostratigraphy | planktic foraminifers, nannofossils | Dowsett (1989a) |
| [DSDP 532 Data](http://www1.ncdc.noaa.gov/pub/data/paleo/contributions_by_author/foley2015/foley2015-dsdp532.txt) | graphic correlation, magnetostratigraphy, astronomical tuning | planktic foraminifers, nannofossils | Dowsett (1989b); Dowsett et al. (1996) |
| [DSDP 541 Data](http://www1.ncdc.noaa.gov/pub/data/paleo/contributions_by_author/foley2015/foley2015-dsdp541.txt) | biochronology | planktic foraminifers, nannofossils | Wilson (1984), Shipboard Scientific Party (1988) |
| [DSDP 546 Data](http://www1.ncdc.noaa.gov/pub/data/paleo/contributions_by_author/foley2015/foley2015-dsdp546.txt) | magnetobiochronology | planktic foraminifers, nannofossils | Hinz et al. (1984) |
| [DSDP 552A Data](http://www1.ncdc.noaa.gov/pub/data/paleo/contributions_by_author/foley2015/foley2015-dsdp552a.txt) | magnetobiochronology, astronomical tuning | planktic foraminifers, nannofossils | Zimmerman et al. (1985); Curry and Miller (1989); (Dowsett and Poore (1990) |
| [DSDP 592 Data](http://www1.ncdc.noaa.gov/pub/data/paleo/contributions_by_author/foley2015/foley2015-dsdp592.txt) | graphic correlation, magnetostratigraphy | planktic foraminifers, nannofossils | Dowsett (1988) |
| [DSDP 603C Data](http://www1.ncdc.noaa.gov/pub/data/paleo/contributions_by_author/foley2015/foley2015-dsdp603c.txt) | magnetobiochronology | planktic foraminifers, nannofossils | Canninga et al. (1986); Ma'alouleh and Moullade (1986); Muza et al. (1986) |
| [DSDP 606 Data](http://www1.ncdc.noaa.gov/pub/data/paleo/contributions_by_author/foley2015/foley2015-dsdp606.txt) | graphic correlation, magnetostratigraphy | planktic foraminifers, nannofossils | Dowsett (1989a); Weaver (1986); Weaver and Clement (1986); Takayama and Sato (1986); Backman and Pestiaux (1986) |
| [DSDP 607 Data](http://www1.ncdc.noaa.gov/pub/data/paleo/contributions_by_author/foley2015/foley2015-dsdp607.txt) | magnetobiochronology, astronomical tuning | planktic foraminifers, nannofossils | Baldauf et al. (1986), Lisiecki and Raymo (2005) |
| [DSDP 608 Data](http://www1.ncdc.noaa.gov/pub/data/paleo/contributions_by_author/foley2015/foley2015-dsdp608.txt) | magnetobiochronology | planktic foraminifers, nannofossils | Baldauf et al. (1986) |
| [DSDP 609B Data](http://www1.ncdc.noaa.gov/pub/data/paleo/contributions_by_author/foley2015/foley2015-dsdp609b.txt) | magnetostratigraphy |  | Baldauf et al. (1986) |
| [DSDP 610A Data](http://www1.ncdc.noaa.gov/pub/data/paleo/contributions_by_author/foley2015/foley2015-dsdp610a.txt) | magnetostratigraphy, astronomical tuning | oxygen isotopes | Baldauf et al. (1986), Lisiecki and Raymo (2005), De Schepper et al. (2013) |
| [ODP 1006A Data](http://www1.ncdc.noaa.gov/pub/data/paleo/contributions_by_author/foley2015/foley2015-odp1006a.txt) | biochronology | planktic foraminifers, nannofossils | Lutz (2010, 2011); Wright and Kroon (2000); Kroon (2000) |
| [ODP 1018A Data](http://www1.ncdc.noaa.gov/pub/data/paleo/contributions_by_author/foley2015/foley2015-odp1018a.txt) | biochronology | planktic foraminifers, nannofossils | Kucera and Kennett (2000) |
| [ODP 1021C Data](http://www1.ncdc.noaa.gov/pub/data/paleo/contributions_by_author/foley2015/foley2015-odp1021c.txt) | magnetostratigraphy |  | Dowsett and Poore (2000) |
| [ODP 1062B Data](http://www1.ncdc.noaa.gov/pub/data/paleo/contributions_by_author/foley2015/foley2015-odp1062b.txt) | magnetobiochronology | planktic foraminifers, nannofossils | Lutz, 2011 |
| [ODP 1063A Data](http://www1.ncdc.noaa.gov/pub/data/paleo/contributions_by_author/foley2015/foley2015-odp1063a.txt) | magnetobiochronology | planktic foraminifers, nannofossils | Lutz, 2011 |
| [ODP 1115B Data](http://www1.ncdc.noaa.gov/pub/data/paleo/contributions_by_author/foley2015/foley2015-odp1115b.txt) | magnetobiochronology, radiometric dating | planktic foraminifers, nannofossils | Seisser (2001); Stoll (2010) |
| [ODP 1236A Data](http://www1.ncdc.noaa.gov/pub/data/paleo/contributions_by_author/foley2015/foley2015-odp1236a.txt) | biochronology, astronomical tuning | nannofossils | Shipboard Scientific Party (2003); Tiedemann et al. (2007) |
| [ODP 1237C Data](http://www1.ncdc.noaa.gov/pub/data/paleo/contributions_by_author/foley2015/foley2015-odp1237c.txt) | astronomical tuning |  | Tiedemann et al. (2007) |
| [ODP 1239B Data](http://www1.ncdc.noaa.gov/pub/data/paleo/contributions_by_author/foley2015/foley2015-odp1239b.txt) | biochronology, astronomical tuning | planktic foraminifers, nannofossils | Tiedemann et al. (2007) |
| [ODP 625B Data](http://www1.ncdc.noaa.gov/pub/data/paleo/contributions_by_author/foley2015/foley2015-odp625b.txt) | biochronology | planktic foraminifers, nannofossils | Joyce et al. (1990) |
| [ODP 646B Data](http://www1.ncdc.noaa.gov/pub/data/paleo/contributions_by_author/foley2015/foley2015-odp646b.txt) | magnetobiochronology | foraminifers | Baldauf et al., 1989 |
| [ODP 659A Data](http://www1.ncdc.noaa.gov/pub/data/paleo/contributions_by_author/foley2015/foley2015-odp659a.txt) | astronomical tuning |  | Tiedemann et al. 1994 |
| [ODP 661A Data](http://www1.ncdc.noaa.gov/pub/data/paleo/contributions_by_author/foley2015/foley2015-odp661a.txt) | magnetobiochronology | planktic foraminifers, nannofossils | Weaver et al., 1989 |
| [ODP 667A Data](http://www1.ncdc.noaa.gov/pub/data/paleo/contributions_by_author/foley2015/foley2015-odp667a.txt) | biochronology | planktic foraminifers, nannofossils | Weaver et al., 1989 |
| [ODP 672A Data](http://www1.ncdc.noaa.gov/pub/data/paleo/contributions_by_author/foley2015/foley2015-odp672a.txt) | biochronology | planktic foraminifers, nannofossils | Shipboard Scientific Party (1988) |
| [ODP 677A Data](http://www1.ncdc.noaa.gov/pub/data/paleo/contributions_by_author/foley2015/foley2015-odp677a.txt) | biochronology | planktic foraminifers, nannofossils | Jenkins and Houghton (1989); Shipboard Scientific Party (1988a) |
| [ODP 704A Data](http://www1.ncdc.noaa.gov/pub/data/paleo/contributions_by_author/foley2015/foley2015-odp704a.txt) | magnetostratigraphy, astronomical tuning |  | Hodell and Venz (1992) |
| [ODP 709C Data](http://www1.ncdc.noaa.gov/pub/data/paleo/contributions_by_author/foley2015/foley2015-odp709c.txt) | magnetobiochronology, astronomical tuning | planktic foraminifers, nannofossils | Shipboard Scientific Party (1988b); Shackleton and Hall (1990); Rio et al. (1990); Karas et al. (2011) |
| [ODP 716B Data](http://www1.ncdc.noaa.gov/pub/data/paleo/contributions_by_author/foley2015/foley2015-odp716b.txt) | magnetobiochronology | planktic foraminifers, nannofossils | Rio et al. (1990) |
| [ODP 722A Data](http://www1.ncdc.noaa.gov/pub/data/paleo/contributions_by_author/foley2015/foley2015-odp722a.txt) | magnetobiochronology | planktic foraminifers, nannofossils | Spaulding et al. (1991) |
| [ODP 747A Data](http://www1.ncdc.noaa.gov/pub/data/paleo/contributions_by_author/foley2015/foley2015-odp747a.txt) | magnetobiochronology | planktic foraminifers, diatom | Harwood et al. (1992) |
| [ODP 751A Data](http://www1.ncdc.noaa.gov/pub/data/paleo/contributions_by_author/foley2015/foley2015-odp751a.txt) | magnetobiochronology | planktic foraminifers, diatom | Harwood et al. (1992); Barron (1996a) |
| [ODP 754A Data](http://www1.ncdc.noaa.gov/pub/data/paleo/contributions_by_author/foley2015/foley2015-odp754a.txt) | magnetobiochronology | planktic foraminifers, nannofossils | Shipboard Scientific Party (1989b) |
| [ODP 757B Data](http://www1.ncdc.noaa.gov/pub/data/paleo/contributions_by_author/foley2015/foley2015-odp757b.txt) | magnetobiochronology | planktic foraminifers, nannofossils | Shipboard Scientific Party, 1989c |
| [ODP 758A Data](http://www1.ncdc.noaa.gov/pub/data/paleo/contributions_by_author/foley2015/foley2015-odp758a.txt) | magnetobiochronology | planktic foraminifers, nannofossils | Farrell and Janecek (1991); Shipboard Scientific Party (1989d); Farrell et al. (1995) |
| [ODP 763A Data](http://www1.ncdc.noaa.gov/pub/data/paleo/contributions_by_author/foley2015/foley2015-odp763a.txt) | magnetobiochronology | planktic foraminifers, nannofossils | Tang (1992); Shipboard Scientific Party (1990) |
| [ODP 769B Data](http://www1.ncdc.noaa.gov/pub/data/paleo/contributions_by_author/foley2015/foley2015-odp769b.txt) | magnetostratigraphy |  | Rangin et al. (1990) |
| [ODP 847C Data](http://www1.ncdc.noaa.gov/pub/data/paleo/contributions_by_author/foley2015/foley2015-odp847c.txt) | magnetobiochronology, GRAPE stratigraphy | planktic foraminifers, nannofossils | Shackleton et al. (1995) |
| [ODP 852B Data](http://www1.ncdc.noaa.gov/pub/data/paleo/contributions_by_author/foley2015/foley2015-odp852b.txt) | magnetobiochronology, GRAPE stratigraphy | planktic foraminifers, nannofossils | Shackleton et al. (1995) |
| [ODP 883BC Data](http://www1.ncdc.noaa.gov/pub/data/paleo/contributions_by_author/foley2015/foley2015-odp883bc.txt) | magnetobiochronology | radiolaria, diatom | Shipboard Scientific Party, 1993; Barron and Gladenkov, 1995 |
| [ODP 887AC Data](http://www1.ncdc.noaa.gov/pub/data/paleo/contributions_by_author/foley2015/foley2015-odp887ac.txt) | magnetobiochronology | diatom | Shipboard Scientific Party (1993); Barron and Gladenkov (1995) |
| [ODP 925B Data](http://www1.ncdc.noaa.gov/pub/data/paleo/contributions_by_author/foley2015/foley2015-odp925b.txt) | astronomical tuning |  | Tiedemann and Franz (1997) |
| [ODP 951A Data](http://www1.ncdc.noaa.gov/pub/data/paleo/contributions_by_author/foley2015/foley2015-odp951a.txt) | magnetobiochronology | planktic foraminifers | Lutz (2011) |
| [ODP 958A Data](http://www1.ncdc.noaa.gov/pub/data/paleo/contributions_by_author/foley2015/foley2015-odp958a.txt) | magnetobiochronology | planktic foraminifers | Lutz (2011) |
| [Rangitikei Data](http://www1.ncdc.noaa.gov/pub/data/paleo/contributions_by_author/foley2015/foley2015-rangitikei.txt) | magnetostratigraphy, astronomical tuning |  | Naish and Wilson (2009) |
| [Yorktown Data](http://www1.ncdc.noaa.gov/pub/data/paleo/contributions_by_author/foley2015/foley2015-yorktown.txt) | magnetobiochronology | planktic foraminifers, nannofossils, ostracods | Dowsett and Wiggs (1992); Cronin (1991a) |

**References**

Barron, J.A. and Gladenkov, A.Y., 1995. Early Miocene to Pleistocene diatom stratigraphy of Leg 145, *Proceedings of the Ocean Drilling Program, Scientific Results*, 145: 3-19.

Barron, J.A., 1996a. Diatom constraints on the position of the Antarctic Polar Front in the middle part of the Pliocene.   *Marine Micropaleontology*, 27:195-213.

Berggren, W. A., D. V. Kent, C. C. Swisher, and M. P. Aubry, 1995. A revised Cenozoic geochronology and chronostratigraphy, in *Geochronology, time scales and global stratigraphic correlation*, vol. 54, edited by W. A. Berggren, D. V. Kent, M. P. Aubry, and J. Hardenbol, pp. 129-212, Society for Sedimentary Geology (Special Publication), Tulsa, OK.

Berggren, W.A., 1972. Cenozoic biostratigraphy and paleobiogeography of the North Atlantic: *Initial Reports of the Deep Sea Drilling Project*, v. 12, n. 14, p. 965-1001.

Berggren, W.A., Kent, D.V. and Van Couvering, J.A., 1985. Neogene geochronology and chronostratigraphy. *The Chronology of the Geological Record*. London, Blackwell Scientific Publications. 211-260.

Cronin, T.M., 1991a. Pliocene shallow water paleoceangraphy of the north Atlantic Ocean based on marine ostracodes. *Quaternary Science Reviews* 10: 175-188.

Dowsett, H.J. and Wiggs, L.B., 1992. Planktonic foraminiferal assemblage of the Yorktown Formation, Virginia, USA. *Micropaleontology* 38: 75-86.

Farrell, J. W., and T. R. Janecek, 1991. Late Neogene Paleoceanography and Paleoclimatology Of The Northeast Indian Ocean (Site 758), in *Proceedings of the Ocean Drilling Program, Scientific Results*, vol. 121, edited by J. Weissel, J. Peirce, E. Taylor, J. Alt et al., pp. 297-355.

Farrell, J. W., S. C. Clemens, and L. P. Gromet, 1995. Improved chronostratigraphic reference curve of late Neogene seawater Sr, *Geology*, 23, 403-406.

Gradstein, F.M., Ogg, J.G. and Schmitz, M., 2012. *The Geologic Time Scale* *2012*, 2-volume set. Elsevier.

Harwood, D.M., Lazarus, D.B., Abelmann, A., Aubry, M.-P., Berggren, W.A., Heider, F., Inokuchi, H., Maruyama, I., McCartney, K., Wei, W., and Wise, S.W., Jr., 1992. Neogene integrated magnetobiostratigraphy of the central Kerguelen Plateau, Leg 120. In Wise, S.W., Jr., Schlich, R., et al., *Proceedings Ocean Drilling Program, Scientific Results*, vol. 120, 1031-1052.

Hodell, D.A. and Venz, K., 1992. Toward a high resolution stable isotopic record of the Southern Ocean during the Pliocene-Pleistocene (4.8-0.8 Ma). *Antarct. Res*. Ser., 56: 265-310.

Jenkins, D. G., and Houghton, S. D., 1989. Late Miocene to Pleistocene planktonic foraminifers from Ocean Drilling Program Site 677, Panama Basin. In Becker, K., Sakai, H., et al., 1989. *Proceedings of the Ocean Drilling Program Scientific Results*, 111: College Station, TX (Ocean Drilling Program), 289-293.

Karas, C., D. Nürnberg, R. Tiedemann, and D. Garbe-Schönberg, 2011. Pliocene Indonesian Throughflow and Leeuwin Current dynamics: Implications for Indian Ocean polar heat flux, *Paleoceanography*, 26, PA2217.

Lisiecki, L. E. and Raymo, M. E., 2005. A Pliocene-Pleistocene stack of 57 globally distributed benthic d^18^O records. *Paleoceanography* 20: 1-17.

Lutz, B.P., 2011. Shifts in North Atlantic planktic foraminifer biogeography and subtropical gyre circulation during the mid-Piacenzian warm period. *Marine Micropaleontology,* 80: 125-149.

Naish, T.R., and Wilson, G.S., 2009. Constraints on the amplitude of Mid-Pliocene (3.6-2.4 Ma) eustatic sea-level fluctuations from the New Zealand shallow-marine sediment record. *Philosophical Transactions of the Royal Society, A*,367:169-187

Rangin, C., Silver, E., et al. 1990. Site 769. *Proceedings of the Ocean Drilling Program, Initial Reports*, 124: 299-342.

Rio, D., E. Fornaciari, and I. Raffi, 1990. Late Oligocene Through Early Pleistocene Calcareous Nannofossils From Western Equatorial Indian Ocean (Leg 115) in *Proceedings of the Ocean Drilling Program, Scientific Results*, vol. 115, edited by R. A. Duncan, J. Backman, L. C. Peterson et al., pp. 175-235.

Shackleton, N. J., Crowhurst, S., Hagelberg, T., Pisias, N. G., and Schneider, D. A., 1995. A new Late Neogene time scale: Application to Leg 138 Sites 73-101, *Proceedings of the Ocean Drilling Program, Scientific Results*.

Shackleton, N., and M.A. Hall, 1990. Pliocene oxygen isotope stratigraphy of Hole 709C, *Proceedings of the Ocean Drilling Program, Scientific Results*, vol. 115**,** edited by R. A. Duncan, J. Backman, L. C. Peterson et al., pp. 529-538.

Shipboard Scientific Party, 1988b. Site 709. *Proceedings of the Ocean Drilling Program, Initial Reports*, vol. 115, edited by J. Backman, R. A. Duncan et al., pp. 459-588.

Shipboard Scientific Party, 1988c. SITE 716, in *Proceedings of the Ocean Drilling Program, Initial Reports*, vol. 115, edited by J. Backman, R. A. Duncan et al., pp. 1005-1073.

Shipboard Scientific Party, 1989a. SITE 722, in *Proceedings of the Ocean Drilling Program, Initial Reports*, vol. 117, edited by W. L. Prell, N. Niitsuma, et al., pp. 255-317.

Shipboard Scientific Party, 1989b. SITE 754, in *Proceedings of the Ocean Drilling Program, Initial Reports*, vol. 121, edited by J. Peirce, J. Weissel, et al., pp. 191-236.

Shipboard Scientific Party, 1989c. SITE 757, in *Proceedings of the Ocean Drilling Program, Initial Reports*, vol. 121, edited by J. Peirce, J. Weissel, et al., pp. 305-358.

Shipboard Scientific Party, 1989d. SITE 758, in *Proceedings of the Ocean Drilling Program, Initial Reports*, vol. 121, edited by J. Peirce, J. Weissel, et al., pp. 359-453.

Shipboard Scientific Party, 1990. SITE 763, in *Proceedings of the Ocean Drilling Program, Initial Reports*, vol. 122, edited by B. U. Haq, U. von Rad et al., pp. 289-352.

Shipboard Scientific Party, 1993. Site 887. In Rea, D.K., Basov, LA., Janecek, T.R., Palmer-Julson, A., et al., *Proceedings of the Ocean Drilling Program, Initial Reports*, 145: College Station, TX (Ocean Drilling Program), 121-208.

Shipboard Scientific Party, 1993a. Site 881. In Rea, D.K., Basov, I.A., Janecek, T.R., Palmer-Julson, A., et al., *Proceedings of the Ocean Drilling Program, Initial Reports*, 145: College Station, TX (Ocean Drilling Program), 37-83.

Shipboard Scientific Party, 1993b. Site 883. In Rea, D.K., Basov, LA., Janecek, T.R., Palmer-Julson, A., et al., *Proceedings of the Ocean Drilling Program, Initial Reports*, 145: College Station, TX (Ocean Drilling Program), 121-208.

Shipboard Scientific Party, 2003. Site 1236. In Mix, A.C., Tiedemann, R., Blum, P., et al., 2003, *Proceedings of the Ocean Drilling Program, Initial Reports*, 202.

Spaulding, S. A., J. Bloemendal, A. Hayashida, J. O. R. Hermelin, K. Kameo, D. Kroon, C. A. Nigrini, T. Sato, T. N. F. Steens, T. Takayama, and S. R. Troelstra, 1991. Magnetostratigraphic and Biostratigraphic Synthesis, Leg 117, Arabian Sea, in *Proceedings of the Ocean Drilling Program, Scientific Results,* vol. 117, edited by W. L. Prell, N. Niitsuma et al., pp. 127-145.

Stoll, D.K., 2010. Mid-Piacenzian sea surface temperature record from ODP Site 1115 in the western equatorial Pacific. *Stratigraphy*, 7(1): 1-6.

Tang, C., 1992. Paleomagnetism Of Cenozoic Sediments In Holes 762B and 763A, Central Exmouth Plateau, Northwest Australia, in *Proceedings of the Ocean Drilling Program, Scientific Results*, vol. 122, edited by U. von Rad, B. U. Haq et al., pp. 717-733.

Tiedemann, R. and Franz, S.O., 1997. Deep-water circulation, chemistry, and terrigenous sediment supply in the equatorial Atlantic during the Pliocene, 3.3-2.6 Ma and 5-4.5 Ma *Proceedings of the Ocean Drilling Program Scientific Results*, 154: 299-318.

Tiedemann, R., Sarnthein, M., and Shackleton, N.J., 1994. Astronomic timescale for the Pliocene Atlantic δ^18^O and dust flux records of Ocean Drilling Program Site 659. *Paleoceanography* 9: 619–638.

Tiedemann, R., Sturm, A., Steph, S., Lund, S.P., and Stoner, J.S., 2007. Astronomically calibrated timescales from 6 to 2.5 Ma and benthic isotope stratigraphies, Sites 1236, 1237, 1239, and 1241. In Tiedemann, R., Mix, A.C., Richter, C., and Ruddiman, W.F. (Eds.), *Proceedings of the Ocean Drilling Program Scientific Results*, 202: College Station, TX (Ocean Drilling Program), 1–69.

Weaver, P.P.E., Backman, J., et al., 1989. Biostratigraphic synthesis, Leg 108, Eastern Equatorial Atlantic. *Proceedings of the Ocean Drilling* *Program, Scientific Results* 108: 455-462.
